# Learning Information Ethical Decision Making With a Simulation Game

**DOI:** 10.3389/fpsyg.2022.933298

**Published:** 2022-06-23

**Authors:** Weijane Lin, Jui-Ying Wang, Hsiu-Ping Yueh

**Affiliations:** ^1^Department of Library and Information Science, National Taiwan University, Taipei, Taiwan; ^2^Graduate School of Comprehensive Human Sciences, University of Tsukuba, Tsukuba, Japan; ^3^Department of Psychology, National Taiwan University, Taipei, Taiwan; ^4^Department of Bio-Industry Communication and Development, National Taiwan University, Taipei, Taiwan

**Keywords:** ethical decision making, information ethics, information literacy, serious game design, simulation game

## Abstract

Taking advantage of the nature of games to deal with conflicting desires through contextual practices, this study illustrated the formal process of designing a situated serious game to facilitate learning of information ethics, a subject that heavily involves decision making, dilemmas, and conflicts between personal, institutional, and social desires. A simulation game with four mission scenarios covering critical issues of privacy, accuracy, property, and accessibility was developed as a situated, authentic and autonomous learning environment. The player-learners were 40 college students majoring in information science and computer science as pre-service informaticists. In this study, they played the game and their game experiences and decision-making processes were recorded and analyzed. The results suggested that the participants’ knowledge of information ethics was significantly improved after playing the serious game. From the qualitative analysis of their behavioral features, including paths, time spans, and access to different materials, the results supported that the game designed in this study was helpful in improving participants’ understanding, analysis, synthesis, and evaluation of information ethics issues, as well as their judgments. These findings have implications for developing curricula and instructions in information ethics education.

## Introduction

The process and scope of information dissemination are expanding along with the rapid development of media and technology. While the access and accessibility to information are improved, in this unique information age, the literacy and ethics of utilizing information have gained importance, especially in the development of information services. Information ethics not only influences people’s behaviors in the production, intermediation, and use of information but also establishes principles for information services governing business and professional practices (Parker et al., 1990; García-Holgado et al., 2021). Related issues, including the privacy, accuracy, property, and accessibility of information, have been widely discussed in previous studies (Mason, 1986; Fallis, 2007; Tavani, 2016), and the global coronavirus pandemic in the past 2 years has greatly sparked public concerns and attention to information privacy and ethics (Zimmer et al., 2020). With the clearer tension between developing and using technology, the enhancement of information literacy education for all people, especially informaticists’ knowledge and skills of information ethics, has received increasing research and practical attention from the industrial and education sectors (Stahl et al., 2016; Eskens, 2020; Fiesler et al., 2020; Stark et al., 2020; Wu et al., 2020). However, the nature, strategies, and pedagogies of ethics education are all parts of a longstanding debate within information and computer science (Saltz et al., 2019).

Ethics is defined as inquiry into the nature and grounds of our moral judgments, standards, and rules of conduct (Taylor, 1974) which focuses on the complex relationships and interactions between people at the social level. Various forms of ethical theories have been discussed and developed for people to explore how to interact with others properly (Arnold and Bowie, 2019). Among these forms, four types of theoretical approaches, namely, consequence-based theories, duty-based theories, rights-based theories, and virtue-based theories, were regarded as critical and fundamental in information ethics education (Fallis, 2007). These four approaches complemented one another through their alternative emphases on different aspects of information ethics, and mastering them facilitated learners’ further understanding of the importance of the codes or principles of ethics. Hunt and Vitell (1986), in their general theory of marketing ethics (GTME), integrated these perspectives and proposed a structural framework to illustrate an ethical decision-making process, which included the two major paths of deontological evaluation and teleological evaluation. Thong and Yap (1998) then conducted an empirical investigation on Hunt and Vitell’s (1986) theory and verified that the ethical decision-making process originally derived from marketing science was also applicable in computing and information contexts. More importantly, it was found that the respondents used different types of information in forming their decisions under different circumstances. However, in their study, the decision-making process was not fully investigated due to the exclusion of several contextual factors. They also reported challenges in presenting actual consequences of respondents’ behavioral intentions due to the limitations of the survey instrument. Other subsequent studies adopted similar methods to validate theoretical models of general or ethical decision-making (Al-Rafee and Cronan, 2006; Moores and Chang, 2006; D’Arcy and Devaraj, 2012) in information-related areas and practices. However, several untested factors, such as organizational (D’Arcy and Devaraj, 2012) and personal contexts (Moores and Chang, 2006), were specifically noted as affecting information professionals’ ethical decisions. As these studies also adopted self-reported, *post hoc* survey instruments, as in Thong and Yap (1998), the relationship linking moral intention to actual behavior remained under-investigated due to the measurement limitations. A more situated and synchronous approach to capture and evaluate the decision-making process is required (Jalali et al., 2019).

## Ethics Education for Information Professionals

As information ethics involves field practices and interactions with others for common well-being, practical concerns have placed great emphasis on issues related to information ethics in the contexts of different professions, types of information, and rapidly changing technologies. Since the 1980s, several professional societies of informaticists, such as the Association for Computing Machinery (Gotterbarn et al., 2018), Institute of Electrical and Electronics Engineers (IEEE, 2020), American Library Association (Witt, 2017; ALA, 2021), and International Federation of Library Associations (IFLA, 2012; Trepanier et al., 2019), have developed their own professional guidelines on ethical conduct to govern the practices of handling information ethics issues. These codes of ethics demonstrated the discipline-specific values and responsibilities of the information professionals and the institutions to society, as library-related associations accentuated issues with providing information services (LAROC, 2002; IFLA, 2012; ALA, 2021), and computer societies highlighted concerns about producing information (ASIS&T Professional Guidelines, 1992; ACM, 2018; IEEE, 2020). On the other hand, these professional conduct guidelines also shared the common inclusion of the four ethical issues of privacy, accuracy, property, and accessibility (Mason, 1986). Nevertheless, due to the highly contextual nature of information ethics (Nissenbaum, 2019), a number of studies have also shown that understanding of the codes is not translated into field practices (Fallis, 2007; McNamara et al., 2018; Saltz et al., 2019).

Although the codes of ethics promulgated by the abovementioned professional societies have often been criticized as oversimplifications of information ethics and therefore as being of little help in solving the practical problems or dilemmas in field practices (Koster, 1992; Fallis, 2007; McMenemy et al., 2014), the development of professional codes of ethics was still regarded as necessary, especially since such codes inform those who are new to the profession about ethical conduct (Rhode, 1992; Gotterbarn et al., 2018). However, the ways different educational institutions and individuals teach ethics are far from homogeneous in terms of the format, content, and structure. In addition, the reviews of school curricula have also found that instruction in professional ethics actually accounts for a rather small percentage of the total information ethics curriculum (Lin and Chou, 2014; Fiesler et al., 2020); most of the pre-service information professionals remained unfamiliar with professional ethics until they entered the workplace. Possible reasons could include school instructors’ perceptions of discrepancies between their disciplinary expertise and the teaching of ethics (Martin, 1997), and as instruction in information ethics was regarded as resource-intensive (Grosz et al., 2019; Fiesler et al., 2020), a lack of structured curriculum and learning mechanisms could have diminished the generalizability of professional ethics instruction (Al-Ansari and Yousef, 2002; Mora et al., 2017).

To address the gap between principle and practice, educational content, resources, and strategies were therefore investigated to supplement learners’ understanding through contextual applications and examples (Skirpan et al., 2018a; Fiesler et al., 2020). Albeit still under-investigated in both the formal and informal education fields (Carbo, 2008; Lin and Chou, 2014; Stahl et al., 2016; Fiesler et al., 2020; García-Holgado et al., 2021), previous instructional practices adopted the common teaching method of lecturing (Lin and Chou, 2014; Fiesler et al., 2020) and also inquiry-based pedagogies such as case-based learning (Fleischmann et al., 2009; Dow et al., 2015), gamification (Brinkman and Miller, 2017), role-playing (Canosa and Lucas, 2008), problem-based learning (Chou et al., 2009; Hou and Tsuei, 2013; Skirpan et al., 2018b), debates (Peace, 2011), and discussions and forums (Chang, 2011; Chang and Chou, 2019) to facilitate learners’ understanding and application of information ethics. However, a critical drawback of these resource-intensive practices, which often involved guidance from mentors or experts, is the limitation of the scalability (Fleischmann et al., 2011; Saltz et al., 2019) and generalizability (Ocholla, 2009) to meet the timely yet scattered, and professional yet interdisciplinary, needs of ethics education in different information practices. As a result, instructional environments and materials that can accommodate structural curriculum and self-directed learning at the same time are greatly needed (Consalvo et al., 2011; Rush, 2014; Mora et al., 2017).

## Serious Games for Information Ethics Education

According to recent reviews of university information and technology ethics courses (Lin and Chou, 2014; Fiesler et al., 2020), the current curriculum of information ethics commonly places great emphasis on the learning outcomes of conceptual skills and on the difficulty of assessing these rather abstract and situated skills. The information ethics courses that were reviewed aimed to develop learners’ understanding of real-world ethical issues, and the instructions intended to improve learners’ decision-making through practicing critical evaluation within different circumstances, perspectives, or consequences. To meet these pedagogical goals and needs, what is needed is a viable and efficient learning environment, which games can provide (Kulman et al., 2011; Rush, 2014). Games have a generally motivational quality, as they induce users to persist on tasks (Prensky, 2003; Kapp, 2012). One specific kind of game that meets the aforementioned instructional needs is simulation games, which have been considered particularly effective in presenting life-like situations to increase psycho-motor skills and in providing opportunities for self-directed evaluation and practices (Hess and Gunter, 2013; Jalali et al., 2019). The basic elements of games, including the goals, challenges, complexity, and simulated scenarios, are also in line with the constructivist learning process (Rosario and Widmeyer, 2009; Kapp, 2012), in which learners are able to develop their knowledge and skills in a situated, self-directed way (Hodhod et al., 2011; Hess and Gunter, 2013).

Game-based learning meets the learning needs of contextual practices in information ethics education (Fallis, 2007; Consalvo et al., 2011; McMenemy et al., 2014). The highly self-directed nature of playing games also provides both novice and expert learners with opportunities to learn and explore at their own paces (Prensky, 2003; Rush, 2014). By using games to teach the specific subject matter of information ethics, previous endeavors mainly focused on the realistic and simulated scenarios afforded by serious games, which could provide personalized experiences for learners and repetitive practice in making ethical decisions (Winter and McCalla, 1999; Hodhod et al., 2009; Lorenzini et al., 2015; Xenos and Velli, 2018). The contexts and rules of the real world can be simulated in such games to provide learners with practical scenarios (Zagal, 2009) for practicing decision making and problem-solving (Hodhod et al., 2011). But compared with the rapidly growing number of studies on gaming media or interfaces (Fu et al., 2022) or the gaming industry (Sotamaa, 2021), empirical research efforts on serious games in disciplinary learning, especially the consideration of professional ethics in information ethics education, remain scarce. Previous empirical studies focused mainly on the general ethical issues, contexts, and skills within experience-based field practices such as hospitals (Lorenzini et al., 2015), the software engineering industry (Xenos and Velli, 2018), and citizen ethics (Hodhod et al., 2011; Bagus et al., 2021). Considering the rapid and extensive changes in information, technology, society, and ethics itself (Capurro, 2006), more up-to-date connections to professional ethics are greatly needed for learning, and more proven design methodologies for instruction are required in professional education and training (McKenzie and McCalla, 2009; Mora et al., 2017).

## Materials and Methods

Motivated by the aforementioned issues, this study intended to design a serious game focused on information ethics for pre-service information professionals’ learning of ethical judgments. To transform the acquisition of ethics theories and professional ethics issues into implicit understanding, analysis, and evaluation, a simulation game was developed as a standalone knowledge acquisition mechanism to support the aforementioned situated learning of information ethics. The instructional effectiveness of the game was investigated based on participants’ learning outcomes, i.e., making well-reasoned ethical decisions. A specific theoretical model of ethical decision-making in the domain of informatics (Hunt and Vitell, 1986; Thong and Yap, 1998) was adopted as the framework for instructional design to support and capture learners’ extended and tightly integrated cognitive learning of information ethics.

After the simulation game was developed, an experiment with pre-service information professionals, namely, college students majoring in information and computer science, was conducted. In the designed game, the player-learners assumed the role of an Internet of Things (IoT) company employee in four mission scenarios of information ethical dilemmas and took responsibility for acting out their roles within the narrative through a structured decision-making process. The game was designed with the Unity engine, and players’ ethical decisions were tied directly to the game simulation *via* changes in quantifiable metrics of their temporal and spatial use logs. The game also recorded participants’ uses and instances of accessing every element as the representations of their decision-making process for further analysis.

In the following sections, the detailed design of the simulation game and the setup of the experiment are described.

### Information Ethics Simulation Game

This study designed a story-driven, first-person role-playing game based on the four major issues of privacy, accuracy, property, and accessibility (PAPA) (Mason, 1986), which are commonly emphasized in the professional codes of ethics in information and computer science (IFLA, 2012; ACM, 2018; IEEE, 2020; ALA, 2021). The story of the game began with an IoT company suffering serious consequences for violating information ethics, and through the game, the player was able to go back in time to relearn the associated concepts and perspectives of information ethics, revisit the environment, and remake the decisions. The game adopted the probing and distributed principles (Rosario and Widmeyer, 2009) to develop a constructivist gaming learning environment instead of plain description and narration. Playing the game required player-learners to actively explore the circumstances and conversations with the stakeholders to retrieve relevant clues and information about the ethical problems and alternatives in order to facilitate users’ attention and reasoning processes.

#### Design of Scenarios

As shown in Table 1, key constructs of the professional ethic codes and the PAPA issues were tailored to the design of the four mission scenarios and alternatives. These scenarios were highly authentic, as they were based on our previous reviews of actual cases, textbook examples, and news stories (Yueh and Lin, 2015). In each mission scenario, two alternatives for action were proposed to the participants for consideration when evaluating each ethical dilemma. The players were able to practice decision-making skills and observe the consequences of a decision iteratively.

**TABLE 1:** Scenarios and alternatives design.

Ethical dilemma	Mission scenarios
Privacy issue	You have been assigned the task of setting up a registration system for a workshop. You used to have an in-house registration system, but recently you’ve seen many other companies use Facebook as an alternative for event registration since it’s more convenient to collect participants’ information. Being the system developer, what will you do?
	Alternative A: Use Facebook Forms for workshop registration.
	Alternative B: Use the original in-house registration system.
Accuracy issue	One day after a regular system update, you have found an error in a customer’s purchase record which could affect his/her rewards points afterward. What will you do?
	Alternative A: Correct the customer’s purchase record.
	Alternative B: Do not amend this record until an official meeting to approve the correction.
Property issue	You got a call from your sister. She needs system management software to complete her school assignment. It just so happens that you have the licensed software, purchased by your company. Will you lend it to your sister?
	Alternative A: Lend the licensed software to your sister.
	Alternative B: Do not lend the licensed software to your sister. Find open-source software for her instead.
Accessibility issue	You are in charge of customer relationship management. However, an inquiry from a senior citizens group has been put on hold by you because senior customers are not the company’s primary consumers. One day when you are trying to handle it, another inquiry from your previous customer comes in. Will you continue to put the original inquiry on hold?
	Alternative A: Continue to put the original inquiry on hold.
	Alternative B: Respond to the inquiries on a first-come, first-served basis.

**FIGURE 1:**
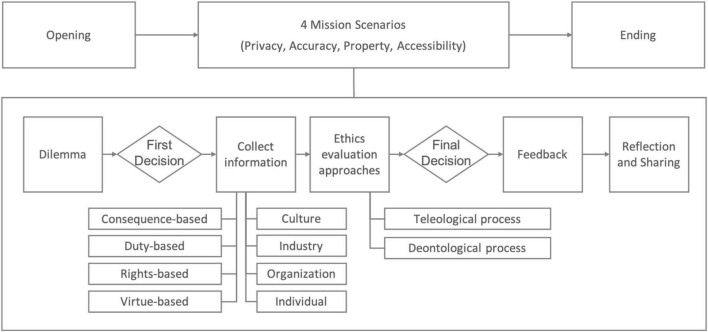
The flow of the game.

Participants experienced the four mission scenarios of privacy, accuracy, property and accessibility in identical sequences (see Figure 1) of encountering an ethical dilemma, making the first decision based on prerequisites and intuition, collecting related information, simulating available evaluation approaches, making the final decision, reviewing the feedback offered by the game, and finally reflecting on the decision.

**FIGURE 2:**
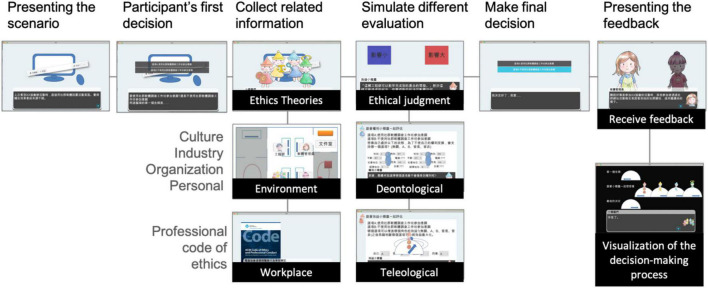
Screenshots of the designed game.

Taking the mission scenario of privacy issues as an example, Figure 2 illustrates each game phase with actual screenshots. Participants were required to practice their ethical thinking and collect information, including the related theories, perspectives, and environmental data, in these situated tasks and contexts. They were also allowed to simulate the consequences of following different evaluation approaches. After they made the final decision, the participants received the rewarding/punitive feedback from the game. Their decision-making process, including their evaluations of theories, environmental factors, and professional conduct, was recorded in its entirety and visualized at the end of each mission scenario for their reflection.

#### Simulation Model in the Game

**FIGURE 3:**
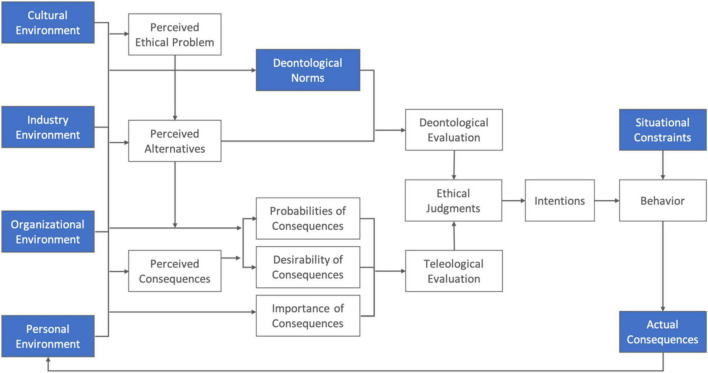
The structure of the simulation model used in the game (Hunt and Vitell, 1986).

This study simulated the ethical decision-making process in game-based ethical analysis training to assist informaticists in coping with new ethical dilemmas presented by information technologies and services. To ensure a comprehensive ethical decision-making process, the theoretical model of the General Theory of Marketing Ethics, or GTME (Hunt and Vitell, 1986), was adopted to design a systemic ethics game. GTME combines the different philosophical considerations of duty (Audi, 1998), rights (Lindblom, 2011), virtue (Koehn, 1995), and utility (Crisp, 1997), and it provides detailed descriptions of important constructs and the interrelationships of individuals’ reasoning processes in making ethical decisions. Possible external factors that affected ethical judgments were included and represented in the game, as shown by the blue markers in Figure 3.

**FIGURE 4:**
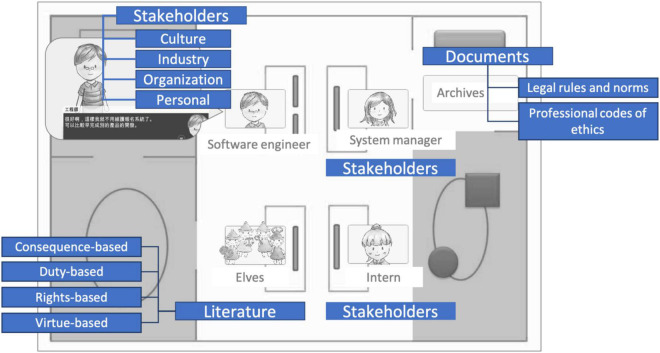
Simulating the factors affecting ethical decisions in the game elements.

As the original model (Hunt and Vitell, 1986) suggested that the ethical decision-making process could be a feedback loop involving manifold factors, such as the types of ethical dilemmas and the environments of culture, industry, organization, and personal experiences, this complex model offered ample alternatives and factors for players to explore in response to different contexts. Therefore, the designed game transformed these factors into corresponding game elements (see Figure 4), including the related documents, literature, and stakeholders that the player-learners encountered during the phase of “collecting information.” The opinions and concerns of stakeholders, such as colleagues, family members, and customers, were provided as external factors to allow participants to perceive consequences from different viewpoints. In addition, to balance the positive and negative consequences, as suggested by Hunt and Vitell (1986), for the two alternatives of A and B in each scenario, the game provided equal numbers of supporting and opposing comments from the surroundings. In addition, legal rules and professional codes of ethics were provided as deontological norms for players’ reference, along with illustrations of critical concepts to help participants to understand the norms and professional conduct. The players were required to evaluate whether the documents affected their ethical decisions. Participants’ time spent on navigating the circumstances and making the decision was also recorded to investigate the efficiency of the decision-making process.

**FIGURE 5:**
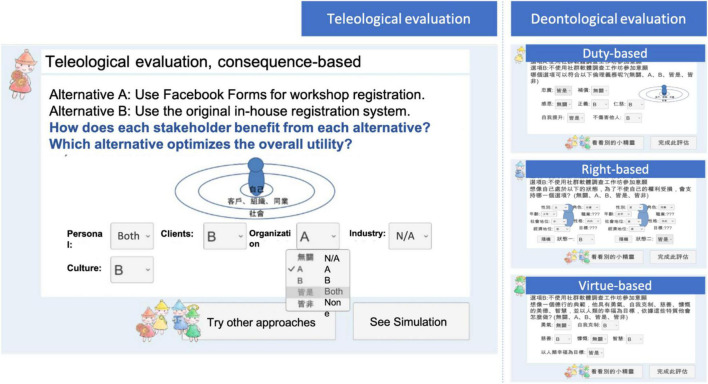
Simulating the consequences of following different evaluation paths.

More importantly, GTME (Hunt and Vitell, 1986) identified an individual’s ethical judgment as a function of both deontological and teleological evaluations. In the deontological path, individuals’ decisions were based on their understanding of the ethical norms along with their duties, rights, or virtues. In the teleological path, individuals made ethical judgments according to their estimation of consequences, including the probability that each consequence would occur, the desirability of each consequence, and the importance of each stakeholder. As shown in Figure 5, to follow the different evaluation paths in the designed game, the player-learners were able to experiment with different evaluation paths and simulate the consequences of decisions made under the selected evaluation, which were based on real events and cases in the past. Furthermore, based on the different philosophical perspectives of ethics, four non-player characters were created as representatives of specific viewpoints. The Elf of Utility provided consequence-based evaluations based on utilitarianism (Crisp, 1997); the Elf of Intuition provided duty-based evaluations based on intuitionism (Audi, 1998); the Elf of Rights provided rights-based evaluations based on Rawl’s theory of rights (Lindblom, 2011), and the Elf of Virtue provided virtue-based evaluations based on Aristotle’s teachings (Koehn, 1995). Critical elements of these theories were explicitly illustrated, and players could simulate the consequences by giving different weights to each element of these theories (see Figure 5).

Hunt and Vitell (1986) claimed in GTME that perceived positive consequences would influence teleological evaluations positively, and teleological evaluations would also affect moral intention directly; therefore, situational constraints such as opportunities to adopt an alternative might result in behaviors that were inconsistent with moral intention. While previous studies (Thong and Yap, 1998; Al-Rafee and Cronan, 2006; Moores and Chang, 2006; D’Arcy and Devaraj, 2012) were challenged by the complexity of GTME due to the difficulty of representing multiple situational elements and consequences with survey instruments, in this current study, the simulation game was adopted to overcome the barriers of representation of constructs and interrelationships and to test the complete model in one instance.

### Experiment Setup

#### Subjects

The subjects for this study were pre-service information professionals, namely, college students majoring in computer science (CS) or library and information science (IS), randomly sampled based on the local statistics of the distribution of majors (Ministry of Education, 2021). The numbers of students majoring in CS (47,196; 3.89%) and IS (41,459; 3.42%) are similar, both exceeding 40,000 students and accounting for nearly 4% of the total population of college students in Taiwan every academic year. The valid number of participants was 40 college students from 7 universities around Taiwan, of which 20 were IT majors from departments of computer science, computer engineering, or electronic engineering, and 20 were IS majors from departments of library and information science, information management, or information communication. All the participants were invited to participate in the experiment and play the information ethics game individually in a laboratory environment. They completed the four mission scenarios in the computer game, and their responses, behaviors, and performances were recorded under their consent.

#### Measures

**FIGURE 6:**
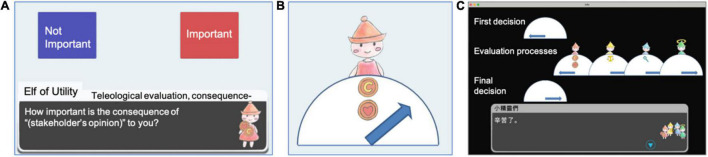
The collection and presentation of game metrics. **(A)** Players’ inputs, session length, and total playtime were stored. **(B)** Visualization of the player’s judgment. **(C)** Summary of player’s inputs for every mission scenario.

In addition to the game metrics (see Figure 6), which recorded participants’ instances of accessing information, paths, and dwelling times on the game elements, a self-developed test for information professionals (Yueh and Lin, 2015) was adopted and distributed before and after the experiment to assess participants’ knowledge of information ethics. The pre-test and post-test each consisted of 16 situational questions with similar themes and identical levels of difficulty regarding ethical dilemmas in the domain of computer and information science. The participants were required to reason and determine if the conduct was informationally ethical. To investigate participants’ gaming experiences, a questionnaire consisting of 12 items was used to inquire about their interactions with the game features and overall satisfaction with the gaming and learning experiences. Example questions for investigating participants’ interaction with the game features were, “Did you notice there was a document on professional codes of ethics in the game? (Yes/No);” and “How helpful to you was the available document in making the final decision? (1–6; Not at all to Extremely).”

## Results and Discussion

All 40 participants completed the four mission scenarios of the game designed in this study. Exactly half of them were IS majors from the departments of library and information science and information management science, and the other half were CS majors from the departments of computer science, computer engineering, and electrical engineering. The sample sizes for graduate and undergraduate students were similar, with 22 graduate students and 18 undergraduates participating. The following sections present the analysis of the participants’ behaviors and learning performances from the perspective of the efficiency and effectiveness of their ethical decision-making processes.

### Player’s Learning Behaviors Captured by the Game Metrics

The total playtime of all participants spent on the designed game was about 50 min (M = 2953.8 s, SD = 907.4 s). The players spent the most time on the mission scenario of privacy (M = 1174.3 s, SD = 465.8 s), and the least time on the mission scenario of accessibility issues (M = 510.5 s, SD = 180.2 s). Participants’ uses of different elves indicated their references to different evaluation methods, and the players spent more time on teleological evaluation with the Elf of Utility (57.2 s), which provided consequence-based information for assessment. Table 2 summarizes the time spent on different areas of the game content.

**TABLE 2:** Total playtime and session length (in seconds).

Items	Total playtime	Opening	S1 Privacy	S2 Accuracy	S3 Property	S4 Accessibility	Ending
**Participants**							
All	2953.8	70.9	1174.3	573.3	545.2	510.5	81.5
CS	2810.3	69.8	1132.7	541.8	512.3	473.2	84.8
IS	3097.2	72.1	1215.8	604.9	578.2	547.9	78.3
**Use of elves**							
Teleological	57.2		125.0	39.1	33.3	31.6	
Utility	57.2		125.0	39.1	33.3	31.6	
Deontological	54.4		110.4	26.4	37.5	29.3	
Duty	54.8		109.5	42.2	37.6	29.8	
Right	59.7		118.3	46.0	42.2	32.1	
Virtue	48.8		103.3	33.1	32.6	26.0	

Table 3 summarizes the time spent for the participants to complete their reasoning and reach judgments in the mission scenarios, whether they were ethical or not. It was found that the participants spent significantly less time making the final decisions than the first decision (M = 5.54 s, SD = 1.43 s) except for the mission scenario of property issues, which suggested that playing the simulation game improved participants’ efficiency of reaching a judgment.

**TABLE 3:** Efficiency of making decisions.

Mission scenarios	Mean	SD	*t*
**S1–Privacy**			
First decision	7.84	5.763	
Final decision	4.683	6.876	2.868**
**S2–Accuracy**			
First decision	3.192	3.123	
Final decision	1.337	0.5	3.883**
**S3–Property**			
First decision	4.695	6.94	
Final decision	2.67	5.497	1.406
**S4–Accessibility**			
First decision	6.435	8.119	
Final decision	2.125	4.971	3.004**

**p < 0.01.

Regarding the quality of the ethical decisions, Table 4 presents a summary of players’ judgments made in every mission scenario and the major basis for their ethical judgments, derived from their uses of and assessments made with every theory-based elf. The game metrics suggested that the participants were able to make ethical judgments when encountering issues of information accuracy, property, and accessibility using both teleological and deontological evaluation. They were more likely to make ethical judgments when they were not biased in any of the evaluation paths and had balanced reference to each theoretical consideration. However, the provision and study of professional ethics and legal rules in the game also helped the participants to make moral judgments by deontological evaluation approaches. The highest average accuracy rate (94%) was achieved by those who made decisions using rights-based evaluation, followed by duty-based (91%), virtue-based (86%), and utility-based (77%). On the other hand, reaching an ethical judgment on privacy-related issues using teleological evaluation with reference to utilitarianism seemed the most challenging for all the participants. As many participants (31/40, 77.5%) pointed out in their reflections on decisions, the conflicts of interest among different stakeholders were so apparent that the participants found it difficult to identify the utilities of the decisions.

**TABLE 4:** Effectiveness of making ethical decisions.

Mission scenarios	Players’ judgment	Basis for ethical judgment							
		Utility-based		Duty-based		Rights-base		Virtue-based	
S1 Privacy	Right	6	9%	22	33%	21	32%	17	26%
	Wrong	18	40%	9	20%	5	11%	13	29%
	Total	24	22%	31	28%	26	23%	30	27%
	Accuracy rate	25%		71%		81%		57%	
S2 Accuracy	Right	25	19%	37	27%	37	27%	36	27%
	Wrong	1	33%	1	33%	0	0%	1	33%
	Total	26	19%	38	28%	37	27%	37	27%
	Accuracy rate	96%		97%		100%		97%	
S3 Property	Right	29	23%	32	25%	35	28%	30	24%
	Wrong	1	25%	1	25%	0	0%	2	50%
	Total	30	23%	33	25%	35	27%	32	25%
	Accuracy rate	97%		97%		100%		94%	
S4 Accessibility	Right	29	22%	36	27%	32	24%	37	28%
	Wrong	3	43%	1	14%	2	29%	1	14%
	Total	32	23%	37	26%	34	24%	38	27%
	Accuracy rate	91%		97%		94%		97%	

**FIGURE 7:**
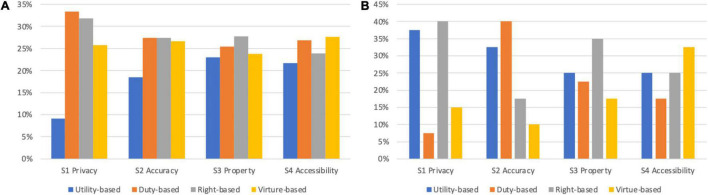
Comparison of participants’ actual and subjective evaluation paths by mission scenario. **(A)** Actual uses of evaluation paths to reach ethical judgments. **(B)** Subjective adoption of evaluation paths and viewpoints.

Interestingly, the game invited every participant to indicate the most decisive theoretical viewpoint in each mission scenario by selecting the most valuable elf, and the results of this subjective selection differed from the above-described behaviors exhibited in the actual decision-making processes captured by the game. Figure 7 presents participants’ actual uses of evaluation paths to reach ethical judgments, as well as their subjective reliance on a specific viewpoint to make a judgment in every mission scenario. While the actual uses of rights and duties for deontological evaluation resulted in ethical judgments, the results of the subjective selection suggested that 30.0% of the participants perceived their decisions to be based primarily on utility, followed by rights (29.4%), duty (21.9%), and finally virtue (18.8%). In addition, participants who were CS and IS majors showed different preferences in the evaluation paths for different ethical issues. For the privacy-related issues, participants majoring in computer science and engineering (CS) exhibited a clear tendency to rely on utilitarianism to make decisions (11/20, 55%), while those who majored in information and library science (IS) counted primarily on rights-based evaluation to make judgments (12/20, 60%).

### Learning Performance of Professional Information Ethics

Aligned with the results of the game metrics, participants’ learning performances, measured by the information ethics test, suggested significant growth in making correct judgments. Table 5 compares participants’ scores in the pre- and post-test. The results suggested that the number of correct ethical judgments in different contexts increased significantly after playing the game. While the IS-majors and CS-majors performed significantly differently on the pre-test (*t* = −4.891, *p* < 0.01), it was found that IS-majors were more aware of ethical issues and considerations due to the human-centered nature of their discipline and professional conduct (LAROC, 2002; Fallis, 2007; IFLA, 2012; ALA, 2021).

**TABLE 5:** Comparison of pre- and post-tests of information ethics.

	Information science					Computer science				
	Pre-test		Post-test			Pre-test		Post-test		
	Mean	SD	Mean	SD	*t*	Mean	SD	Mean	SD	*t*
Privacy	7.5	1.701	9.1	2.49	−3.31*	6.15	1.785	7.05	1.932	−2.651*
Accuracy	7.55	2.259	10.05	1.932	−4.544*	5.95	2.395	9.4	1.569	−5.705*
Property	7.4	3.604	9.2	2.648	−2.155*	5.85	2.996	7.05	2.724	−1.955
Accessibility	1.1	3.919	6.05	3.348	−5.777*	0.7	2.155	4.1	3.076	−4.587*
Total score	48.4	13.732	64.2	14.34	−4.981*	40	12.482	53.25	12.904	−5.229*

*p < 0.05.

On the other hand, as the game provided conceptual knowledge about ethics theories and professional ethics in the form of resource documents and non-player characters (elves), at the end of the game, all participants were asked to respond to four short-answer questions about their understanding of the four theoretical viewpoints. The qualitative analysis of the participants’ answers showed that the majority of the participants established a basic understanding of the four major ethics theories, and they were able to make well-reasoned arguments and see multiple perspectives to approach information ethics issues. Specifically, 97.5% of the participants correctly described consequence-based evaluation based on utilitarianism, and 80.0, 75, and 65% appropriately interpreted rights-based, duty-based, and virtue-based viewpoints, respectively.

### Learning Experiences of Information Ethics With the Simulation Game

The player-learner participants gave high appraisals on the simulation game in terms of its interactions and interface. As shown in Table 6, using a six-point scale, participants regarded playing the simulation game as the most helpful to raise their awareness of ethical issues and multiple perspectives (M = 5.33/6), improve their understanding of information ethics (M = 5.13/6) and decision-making skills in the workplaces (M = 5.01/6), and increase their confidence in dealing with ethical issues in the future (M = 4.8/6). Participants’ evaluations of the game features obtained from both the quantitative survey and qualitative interviews suggested that the participants regarded the game elements of four mission scenarios (M = 5.0/6) and different stakeholders’ concerns (M = 5.0/6) as the most helpful. These two elements, which were the core of this simulation game, were originally designed to reinforce the representation of authentic situations and external factors that would affect players’ ethical judgments. The participants also appreciated the provision and visualization of players’ first and final decisions (M = 4.6/6), for this review helped them to associate their ethical judgments with their intentions. The four elves (M = 4.3/6) functioned to guide the player-learners to discover the alternative perspectives behind the ethical decisions which they had not known or noticed before, and in the interviews, several participants reflected on their motivation in investigating how ethical decisions might affect others in terms of their rights, duties, and utilities. Finally, participants’ affirmation of the integration of professional ethical conduct with the mission scenarios (M = 4.0/6) also provided empirical support for the design intention of this simulation game to translate deontological norms into field practices (McNamara et al., 2018; Saltz et al., 2019). As the participants became aware of the importance of a professional code of ethics, several interviewees mentioned they would be able to deal with ethical issues earlier in their future practices by referencing the relevant professional conduct. Some even considered developing their own codes of ethics within their organizations as necessary to guide customized professional conduct within the specific organizational environment.

**TABLE 6:** Evaluation of the simulation game.

Evaluation	IS (*N* = 20)		CS (*N* = 20)	
Game interaction	M	SD	M	SD
Playing the game improved my knowledge of information ethics	5.05/6	0.887	5.2/6	0.951
Playing the game improved my awareness of ethical issues and perspectives	5.4/6	0.883	5.25/6	0.91
Playing the game improved my future decision making in the workplaces	5.1/6	0.912	4.95/6	1.191
Playing the game improved my confidence in dealing with ethical issues in the future	4.6/6	1.046	4.9/6	0.968
**Game interface features**				
The four mission scenarios	5.17/6	1.1	4.83/6	1.25
Resource documents introducing the four theoretical issues	4.18/6	1.43	3.63/6	1.34
Professional ethical conduct	4.53/6	1.31	3.53/6	1.5
Stakeholders’ concerns	5/6	1.03	4.85/6	1.18
Visualization of my judgments	3.29/6	1.69	2.9/6	1.52
The provision of alternative perspectives by the elves	4.39/6	1.29	4.3/6	1.17
Provision and visualization of first and final decisions	4.83/6	1.3	4.45/6	1.32
Reflecting on the decision and sharing my thoughts with others	3.82/6	1.63	3.72/6	1.49

## Conclusion

This study simulated the ethical decision-making process in game-based ethical analysis training to assist informaticists in coping with new ethical dilemmas brought about by information technologies and services. Taking advantage of the nature of the game to deal with conflicting desires, this study designed and developed a simulation game as an authentic and autonomous learning environment for teaching pre-service information professionals about information ethics, a subject that heavily involves decision making, dilemmas, and conflicts between personal and social desires. Using the theoretical framework of GTME (Hunt and Vitell, 1986) as the simulation model, which encompassed a comprehensive process for ethical decision making, the game elements of stories, characters, scenarios, and tasks were developed accordingly. A total of 40 pre-service information professionals were recruited to evaluate whether the game mechanism was compatible with modern field practices and whether the simulation game assisted their learning of information ethics. According to the results of the player-learners’ evaluations, the theoretical propositions concerning simulation in the design of the game-based ethical analysis training were confirmed. Both the efficiency and effectiveness of the participants’ decision-making process were improved. They were able to apply rules, consider consequences, see multiple perspectives, and perceive issues within the game, and they performed better on making ethical judgments after playing it. Participants’ overall satisfaction with the learning experiences proved the feasibility of game-based training for information ethics.

Methodologically, this study contributes to the field studies of decision science by adopting the mechanism of a simulation game to represent the complex situational elements and consequences of the ethical decision-making process, which in the past was a challenge due to the limitations of the survey instruments (Thong and Yap, 1998; Al-Rafee and Cronan, 2006; Moores and Chang, 2006; D’Arcy and Devaraj, 2012). The designated simulation game was able to capture and test the complete decision-making models of every player-learner in one instance, as supported by the research findings of the differences in the valid uses of evaluation paths from self-perceived preferences. The results offered evidence supporting the use of the game designed in this study as a complete learning environment for both learning and evaluation of information ethics. While several limitations, including the one-off session experiment and relatively small sample size, should be noted, the systematic and empirical investigation conducted in this study on supporting information professionals’ ethical decision-making process through simulation games could serve as a basis for designing assessment tools in the future, as it has found critical elements for designing serious games. Based on the findings of the study, the designed serious game that accommodates the structural curriculum within university teaching and students’ self-directed learning can serve as a useful learning resource for pre-service information professionals. The game contains comprehensive and critical information ethics issues, which can not only be used as supplementary materials in information engineering and science classes but provides learners with sufficient opportunities for contextual practices. As this study offers a useful framework to inform the design of game-based ethical analysis training for both instructional and self-directed learning uses, given the highly contemporary nature of information ethics, it is also suggested that further studies or practices could extend or incorporate more contexts, cases, or examples in the future.

## Data Availability Statement

The raw data supporting the conclusions of this article will be made available by the authors, without undue reservation.

## Ethics Statement

Ethical review and approval was not required for the study on human participants in accordance with the local legislation and institutional requirements. The patients/participants provided their written informed consent to participate in this study.

## Author Contributions

WL: conceptualization, methodology, visualization, investigation, and writing—original draft. J-YW: software and investigation. H-PY: methodology, funding acquisition, and writing—reviewing and editing. All authors contributed to the article and approved the submitted version.

## Conflict of Interest

The authors declare that the research was conducted in the absence of any commercial or financial relationships that could be construed as a potential conflict of interest.

## Publisher’s Note

All claims expressed in this article are solely those of the authors and do not necessarily represent those of their affiliated organizations, or those of the publisher, the editors and the reviewers. Any product that may be evaluated in this article, or claim that may be made by its manufacturer, is not guaranteed or endorsed by the publisher.

## References

[B1] ACM (2018). *ACM Code of Ethics and Professional Conduct.* Available Online at: https://www.acm.org/code-of-ethics (accessed February 18, 2022).

[B2] ALA (2021). *ALA Code of Ethics Adopted at the 1939 Midwinter Meeting by the ALA Council; Amended June 30, 1981; June 28, 1995; January 22, 2008; and June 29, 2021.* Available Online at: https://www.ala.org/tools/ethics (accessed February 18, 2022).

[B3] Al-Ansari H. Yousef N. (2002). Coverage of competencies in the curriculum of information studies: an international perspective. *Educ. Inf.* 20 199–215. 10.3233/EFI-2002-203-403

[B4] Al-Rafee S. Cronan T. P. (2006). Digital piracy: factors that influence attitude toward behavior. *J. Bus. Ethics* 63 237–259. 10.1007/s10551-005-1902-9

[B5] Arnold D. G. Bowie N. E. (2019). *Ethical theory and Business.* Cambridge: Cambridge University Press. 10.1017/9781108386128

[B6] ASIS&T Professional Guidelines (1992). Available Online at: https://www.asist.org/about/asist-professional-guidelines/ (accessed February 18, 2022).

[B7] Audi R. (1998). Moderate intuitionism and the epistemology of moral judgment. *Ethical Theory Moral Pract.* 1 15–44. 10.1023/A:1009904328068

[B8] Bagus D. Setiawan K. Arisaputra P. Harefa J. Chowanda A. (2021). Designing serious games to teach ethics to young children. *Procedia Comput. Sci.* 179 813–820. 10.1016/j.procs.2021.01.069 29773528

[B9] Brinkman B. Miller K. W. (2017). “The code of ethics quiz show,” in *Proceedings of the 2017 SIGCSE Technical Symposium on Computer Science Education*, (New York, NY: ACM). 10.1145/3017680.3017803

[B10] Canosa R. L. Lucas J. M. (2008). “Mock trials and role-playing in computer ethics courses,” in *Proceedings of the 39th SIGCSE Technical Symposium on Computer Science Education*, (New York, NY: ACM). 10.1145/1352135.1352187

[B11] Capurro R. (2006). Towards an ontological foundation of information ethics. *Ethics Inf. Technol.* 8 175–186. 10.1007/s10676-006-9108-0

[B12] Carbo T. (2008). Ethics education for information professionals. *J. Libr. Adm.* 47 5–25. 10.1080/01930820802186324

[B13] Chang C. L.-H. (2011). The effect of an information ethics course on the information ethics values of students–a Chinese guanxi culture perspective. *Comput. Hum. Behav.* 27 2028–2038. 10.1016/j.chb.2011.05.010

[B14] Chang C.-M. Chou C. (2019). Development of instructions for e-character education for the sixth graders. *Curric. Instr. Q.* 22 1–25.

[B15] Chou C. Wu H.-C. Chen Y.-L. Wang M.-H. (2009). The “information literacy and ethics” curriculum for college general education: rational, topics, instructional design strategies, and implementation. *Univ. Libr. Q.* 13 24–44.

[B16] Consalvo M. Costikyan G. Davidson D. Fortugno N. Shaenfield D. Vigeant P. (2011). “Quick takes on ethics and games voices from industry and academia,” in *Designing Games for Ethics: Models, Techniques and Frameworks*, eds Karen S. David G. (Pennsylvania, PA: IGI Global), 1–18. 10.4018/978-1-60960-120-1.ch001

[B17] Crisp R. (1997). *Routledge Philosophy Guidebook to Mill on Utilitarianism.* Hove: Psychology Press.

[B18] D’Arcy J. Devaraj S. (2012). Employee misuse of information technology resources: testing a contemporary deterrence model. *Decis. Sci.* 43 1091–1124. 10.1111/j.1540-5915.2012.00383.x

[B19] Dow M. J. Boettcher C. A. Diego J. F. Karch M. E. Todd-Diaz A. Woods K. M. (2015). Case-based learning as pedagogy for teaching information ethics based on the Dervin sense-making methodology. *J. Educ. Libr. Inf. Sci.* 56 141–157. 10.12783/issn.2328-2967/56/2/2

[B20] Eskens S. (2020). The personal information sphere: an integral approach to privacy and related information and communication rights. *J. Assoc. Inf. Sci. Technol.* 71 1116–1128. 10.1002/asi.24354 32984437 PMC7496843

[B21] Fallis D. (2007). Information ethics for twenty-first century library professionals. *Library Hi Tech* 25 23–36. 10.1108/07378830710735830 9061155

[B22] Fiesler C. Garrett N. Beard N. (2020). “What do we teach when we teach tech ethics? A syllabi analysis,” in *Proceedings of the 51st ACM Technical Symposium on Computer Science Education*, Portland, OR. 10.1145/3328778.3366825

[B23] Fleischmann K. R. Robbins R. W. Wallace W. A. (2009). Designing educational cases for intercultural information ethics: the importance of diversity, perspectives, values, and pluralism. *J. Educ. Libr. Inf. Sci.* 50 4–14.

[B24] Fleischmann K. R. Robbins R. W. Wallace W. A. (2011). Information ethics education for a multicultural world. *J. Inf. Syst. Educ.* 22 191–202.

[B25] Fu Y. Hu Y. Sundstedt V. (2022). A systematic literature review of virtual, augmented, and mixed reality game applications in healthcare. *ACM Trans. Comput. Healthc.* 3 1–27. 10.1145/3472303 33011296

[B26] García-Holgado A. García-Peñalvo F. J. Therón R. Vázquez-Ingelmo A. Gamazo A. González-González C. S. (2021). “Development of a SPOC of computer ethics for students of computer science degree,” in *Proceedings of the XI JICV 2021. XI International Conference on Virtual Campus (Salamanca, Spain, September 30th – October 1st, 2021)*, eds García-Holgado A. García-Peñalvo F. J. González-González C. S. Infante-Moro A. Infante-Moro J. C. (New York, NY: IEEE).

[B27] Gotterbarn D. Brinkman B. Flick C. Kirkpatrick M. S. Miller K. Vazansky K. (2018). *ACM Code of Ethics and Professional Conduct.* New York, NY: ACM.

[B28] Grosz B. J. Grant D. G. Vredenburgh K. Behrends J. Hu L. Simmons A. (2019). Embedded EthiCS: integrating ethics across CS education. *Commun. ACM* 62 54–61. 10.1145/3330794

[B29] Hess T. Gunter G. (2013). Serious game-based and nongame-based online courses: learning experiences and outcomes. *Br. J. Educ. Technol.* 44 372–385. 10.1111/bjet.12024 33949956

[B30] Hodhod R. Cairns P. Kudenko D. (2011). “Innovative integrated architecture for educational games: challenges and merits,” in *Transactions on Edutainment V*, eds Pan Z. Cheok A. D. Müller W. Yang X. (Berlin: Springer), 1–34. 10.1007/978-3-642-18452-9_1

[B31] Hodhod R. Kudenko D. Cairns P. (2009). “AEINS: adaptive educational interactive narrative system to teach ethics,” in *Proceedings of the 14th International Conference on Artificial Intelligence in Education Workshops AIED 2009*, Brighton.

[B32] Hou C.-H. Tsuei M.-P. (2013). A study of developing the problem-based learning system on information literacy and ethic curriculum for fifth-grade students: applying on the internet copyright courses. *Res. Educ. Commun. Technol.* 104 17–36.

[B33] Hunt S. D. Vitell S. (1986). A general theory of marketing ethics. *J. Macromarketing* 6 5–16. 10.1177/027614678600600103

[B34] IEEE (2020). *IEEE Code of Ethics.* Available Online at: https://www.ieee.org/about/corporate/governance/p7-8.html (accessed February 18, 2022).

[B35] IFLA (2012). *IFLA Code of Ethics for Librarians and Other Information Workers (Long Version).* Available Online at: https://repository.ifla.org/handle/123456789/1850 (accessed February 18, 2022).

[B36] Jalali M. S. Siegel M. Madnick S. (2019). Decision-making and biases in cybersecurity capability development: evidence from a simulation game experiment. *J. Strateg. Inf. Syst.* 28 66–82. 10.1016/j.jsis.2018.09.003

[B37] Kapp K. M. (2012). *The Gamification of Learning and Instruction: Game-Based Methods and Strategies for Training and Education.* Hoboken, NJ: John Wiley & Sons. 10.1145/2207270.2211316

[B38] Koehn D. (1995). A role for virtue ethics in the analysis of business practice. *Bus. Ethics Q.* 5 533–539. 10.2307/3857397

[B39] Koster G. E. (1992). Ethics in reference service: codes, case studies, or values? *Ref. Serv. Rev.* 20 71–80. 10.1108/eb049148

[B40] Kulman R. Stoner G. Ruffolo L. Marshall S. Slater J. Dyl A. (2011). “Teaching executive functions, self-management, and ethical decision-making through popular videogame play,” in *Designing Games for Ethics: Models, Techniques and Frameworks*, eds Schrier K. Gibson D. (Pennsylvania, PA: IGI Global), 193–207. 10.4018/978-1-60960-120-1.ch013

[B41] LAROC (2002). *Code of Professional Ethics for Librarians in Taiwan.* Available Online at: http://www.lac.org.tw/law/12 (accessed February 18, 2022).

[B42] Lin C.-H. Chou C. (2014). Ethics curricula of the information science departments in Taiwanese universities and colleges. *J. Res. Educ. Sci.* 59 197–228.

[B43] Lindblom L. (2011). The structure of a Rawlsian theory of just work. *J. Bus. Ethics* 101 577–599. 10.1007/s10551-011-0740-1

[B44] Lorenzini C. Faita C. Barsotti M. Carrozzino M. Tecchia F. Bergamasco M. (2015). “ADITHO–a serious game for training and evaluating medical ethics skills,” in *Proceedings of the International Conference on Entertainment Computing*, Vol. 9353 eds Chorianopoulos K. Divitini M. Baalsrud Hauge J. Jaccheri L. Malaka R. (Cham: Springer). 10.1007/978-3-319-24589-8_5

[B45] Martin C. D. (1997). “The case for integrating ethical and social impact into the computer science curriculum,” in *Proceedings of the Supplemental Conference on Integrating Technology into Computer Science Education: Working Group Reports and Supplemental Proceedings*, (New York, NY: ACM). 10.1145/266057.266131

[B46] Mason R. O. (1986). Four ethical issues of the information age. *MIS Q.* 10 5–12. 10.2307/248873

[B47] McKenzie A. McCalla G. (2009). “Serious games for professional ethics: an architecture to support personalization,” in *Proceedings of the 14th International Conference on Artificial Intelligence in Education Workshops AIED 2009*, Brighton.

[B48] McMenemy D. Poulter A. Burton P. (2014). *A Handbook of Ethical Practice: a Practical Guide to Dealing with Ethical Issues in Information and Library Work.* Amsterdam: Elsevier.

[B49] McNamara A. Smith J. Murphy-Hill E. (2018). “Does ACM’s code of ethics change ethical decision making in software development?,” in *Proceedings of the 2018 26th ACM Joint Meeting on European Software Engineering Conference and Symposium on the Foundations of Software Engineering*, Tallinn. 10.1145/3236024.3264833

[B50] Ministry of Education (2021). *Annual Report of Education Statistics.* Taipei: Ministry of Education.

[B51] Moores T. T. Chang J. C.-J. (2006). Ethical decision making in software piracy: initial development and test of a four-component model. *MIS Q.* 30 167–180. 10.2307/25148722

[B52] Mora A. Riera D. González C. Arnedo-Moreno J. (2017). Gamification: a systematic review of design frameworks. *J. Comput. High. Educ.* 29 516–548. 10.1007/s12528-017-9150-4

[B53] Nissenbaum H. (2019). Contextual integrity up and down the data food chain. *Theo. Inquiries Law* 20 221–256. 10.1515/til-2019-0008

[B54] Ocholla D. (2009). Information ethics education in Africa. Where do we stand? *Int. Inf. Libr. Rev.* 41 79–88. 10.1080/10572317.2009.10762802

[B55] Parker D. B. Swope S. Baker B. N. (1990). *Ethical Conflicts: in Information and Computer Science, Technology, and Business.* Wellesley, MA: QED Information Sciences, Inc.

[B56] Peace A. G. (2011). Using debates to teach information ethics. *J. Inf. Syst. Educ.* 22 233–238.

[B57] Prensky M. (2003). Digital game-based learning. *Comput. Entertain.* 1:21. 10.1145/950566.950596

[B58] Rhode D. L. (1992). Ethics by the pervasive method. *J. Legal Educ.* 42 31–56.

[B59] Rosario R. A. Widmeyer G. R. (2009). An exploratory review of design principles in constructivist gaming learning environments. *J. Inf. Syst. Educ.* 20:289.

[B60] Rush L. (2014). Learning through play, the old school way: teaching information ethics to millennials. *J. Libr. Innov.* 5 1–14.

[B61] Saltz J. Skirpan M. Fiesler C. Gorelick M. Yeh T. Heckman R. (2019). Integrating ethics within machine learning courses. *ACM Trans. Comput. Educ.* 19 1–26. 10.1145/3341164

[B62] Skirpan M. Beard N. Bhaduri S. Fiesler C. Yeh T. (2018a). “Ethics education in context: a case study of novel ethics activities for the CS classroom,” in *Proceedings of the 49th ACM Technical Symposium on Computer Science Education*, (New York, NY: ACM). 10.1145/3159450.3159573

[B63] Skirpan M. Cameron J. Yeh T. (2018b). “Quantified self: an interdisciplinary immersive theater project supporting a collaborative learning environment for CS ethics,” in *Proceedings of the 49th ACM Technical Symposium on Computer Science Education*, (New York, NY: ACM). 10.1145/3159450.3159574

[B64] Sotamaa O. (2021). Studying game development cultures. *Games Cult.* 16 835–854. 10.1177/15554120211005242

[B65] Stahl B. C. Timmermans J. Mittelstadt B. D. (2016). The ethics of computing: a survey of the computing-oriented literature. *ACM Comput. Surv.* 48 1–38. 10.1145/2871196

[B66] Stark L. Stanhaus A. Anthony D. L. (2020). “I don’t want someone to watch me while I’m working”: gendered views of facial recognition technology in workplace surveillance. *J. Assoc. Inf. Sci. Technol.* 71 1074–1088. 10.1002/asi.24342

[B67] Tavani H. T. (2016). *Ethics and Technology: Controversies, Questions, and Strategies for Ethical Computing.* Hoboken, NJ: John Wiley & Sons.

[B68] Taylor P. W. (1974). Principles of ethics: an introduction. *Teach. Philos.* 1 215–216. 10.5840/teachphil19751267

[B69] Thong J. Y. Yap C.-S. (1998). Testing an ethical decision-making theory: the case of softlifting. *J. Manag. Inf. Syst.* 15 213–237. 10.1080/07421222.1998.11518203

[B70] Trepanier C. Shiri A. Samek T. (2019). An examination of IFLA and data science association ethical codes. *IFLA J.* 45 289–301. 10.1177/0340035219849614

[B71] Winter M. McCalla G. (1999). “The emergence of student models from an analysis of ethical decision making in a scenario-based learning environment,” in *UM99 User Modeling* CISM International Centre for Mechanical Sciences, Vol. 407 ed. Kay J. (Vienna: Springer), 265–274. 10.1007/978-3-7091-2490-1_26

[B72] Witt S. (2017). The evolution of privacy within the American library association, 1906–2002. *Libr. Trends* 65 639–657. 10.1353/lib.2017.0022 34409987

[B73] Wu P. F. Vitak J. Zimmer M. T. (2020). A contextual approach to information privacy research. *J. Assoc. Inf. Sci. Technol.* 71 485–490. 10.1002/asi.24232

[B74] Xenos M. Velli V. (2018). “A serious game for introducing software engineering ethics to university students,” in *Proceedings of the International Conference on Interactive Collaborative Learning*, Vol. 916 eds Auer M. Tsiatsos T. (Cham: Springer). 10.1007/978-3-030-11932-4_55

[B75] Yueh H.-P. Lin W. (2015). Using the Hypothetical-Deductive Model to Build a Web-Based Workplace Ethics Learning Platform to Develop Students’ Ethic Reasoning Ability: Perspective of Information Workers National Science Council Report No. NSC102-2511-S002-007-MY2.

[B76] Zagal J. P. (2009). “Ethically notable videogames: moral dilemmas and gameplay,” in *Proceedings of the DiGRA International Conference: Breaking New Ground: Innovation in Games, Play, Practice and Theory*, Uxbridge.

[B77] Zimmer M. Vitak J. Wu P. (2020). Editorial introduction: “information privacy in the digital age”. *J. Assoc. Inf. Sci. Technol.* 71 997–1001. 10.1002/asi.24394

